# Impact of the Order of Legendre Polynomials in Random Regression Model on Genetic Evaluation for Milk Yield in Dairy Cattle Population

**DOI:** 10.3389/fgene.2020.586155

**Published:** 2020-11-05

**Authors:** Jianbin Li, Hongding Gao, Per Madsen, Rongling Li, Wenhao Liu, Peng Bao, Guanghui Xue, Yundong Gao, Xueke Di, Guosheng Su

**Affiliations:** ^1^Dairy Cattle Research Center, Shandong Academy of Agricultural Sciences, Jinan, China; ^2^Center for Quantitative Genetics and Genomics, Aarhus University, Tjele, Denmark; ^3^Shandong OX Livestock Breeding Industry Co., Ltd, Jinan, China; ^4^Linqing Rutai Animal Husbandry Co., Ltd, Liaocheng, China

**Keywords:** genetic evaluation, genetic parameters, random regression, test-day records, legendre polynomials

## Abstract

The random regression test-day model has become the most commonly adopted model for routine genetic evaluations in dairy populations, which allows accurately accounting for genetic and environmental effects over lactation. The objective of this study was to explore appropriate random regression test-day models for genetic evaluation of milk yield in a Holstein population with a relatively small size, which is the common situation in regional or independent breeding companies to preform genetic evaluation. Data included 419,567 test-day records from 54,417 cows from the first lactation. Variance components and breeding values were estimated using a random regression test-day model with different orders (from first to fifth) of Legendre polynomials (LP) and accounted for homogeneous or heterogeneous residual variance across the lactation. Models were compared based on Akaike information criterion (AIC), Bayesian information criterion (BIC), and predictive ability. In general, models with a higher order of LP showed better goodness of fit based on AIC and BIC values. However, models with third, fourth, and fifth order of LP led to similar estimates of genetic parameters and predictive ability. Models with assumption of heterogeneous residual variances achieved better goodness of fit but yielded similar predictive ability compared with those with assumption of homogeneous residual variances. Therefore, a random regression model with third order of LP is suggested to be an appropriate model for genetic evaluation of milk yield in local Chinese Holstein populations.

## Introduction

The random regression test-day model has been widely used in genetic evaluation for production traits in dairy cattle and has many advantages, including more accurately accounting for genetic and environment effects at different stages of lactation, thus resulting in more reliable genetic evaluation (Schaeffer and Dekkers, 1994; Jamrozik et al., 1997b; Schaeffer et al., 2000; Jensen, 2001; Schaeffer, 2004). The test-day model is significantly better than the lactation model (using full and extended 305 days lactation records) with 2–3% increase in accuracy for bulls and 6–8% for cows for milk yield first lactation (Kistemaker, 1997). In addition, the test-day model allows predicting estimated breeding value (EBV) for each test-day, each particular period, or the full lactation (305 days) and persistency (Jamrozik et al., 1997b).

The functions generally used to model the lactation curve include Woods’ model (Wood, 1967), Wilmink’s function (Wilmink, 1987), spline function (White et al., 1999), and Legendre polynomial (LP) function (Kirkpatrick et al., 1990). Because of differences in production environments and management systems, optimal functions for test-day models in different countries may be different (Reinhardt et al., 2002; Mrode et al., 2003). Several studies have shown that LPs performed well in random regression test-day models, but there is no “gold standard” reported in literature on choosing optimal order of LPs in the model, and the choice of the order of fit is highly dependent on the practical data structures and populations. For example, a fourth order LP (LP4) was used for national genetic evaluations in Canada and Italy, and a fifth order LP (LP5) was used in the United Kingdom (Muir et al., 2007). The joint Nordic test-day model was a multivariate model for milk, protein, and fat in lactation one to three (in total, nine traits), the genetic and permanent environmental effects were modeled by second order LP and an exponential term *e*^−0.04×*DIM*^ (Lidauer et al., 2015).

The dairy herd improvement (DHI) project was first implemented in four provinces in the 1990s in China. In 2006, the Ministry of Agriculture of China approved a project to promote the DHI project in eight provinces where there were many large dairy populations (Miglior et al., 2009). Recently, the project has been expanded to 25 provinces in China, where provincial DHI laboratories and data centers have been established (Yearbook, 2016), and there were about 700,000 cows recording milk production in China each year. Many organizations and companies prefer to conduct genetic evaluation based on their own populations by considering the differences among populations. In general, these populations are relatively small in size. Therefore, an appropriate parametric function that can sufficiently account for the lactation curve and avoid overparameterization is critical for relatively small populations when using a random regression test-day model to estimate genetic parameters and breeding values. Relatively little research has been placed on the impact of parametric functions on genetic evaluation in a numerically small size dairy cattle population.

The aim of this study was to investigate the impact of the order of LPs on estimation of genetic parameters and prediction of breeding values for the milk yield trait and find appropriate orders of LPs in random regression test-day models for genetic evaluation in numerically small cattle populations, such as genetic evaluations at the regional level in China.

## Materials and Methods

### Data

Data were obtained from the database at the Dairy Cattle Research Centre DHI Lab, Shandong Academy of Agricultural Sciences. First were lactation records from 2004–2015 that fulfill the following criteria: ages at calving between 20 and 38 months, daily milk yield between 5 and 80 kg, days in milk (DIM) between 5 and 305, and cows with at least three test-day records were extracted. The final data set consisted of 419,567 test-day records from 54,417 cows. The number of records in each DIM class ranged from 576 to 1768. Descriptive statistics of the data are presented in Table 1. The traced pedigree included 104,884 individuals.

**TABLE 1:** Number of records (*N*), mean, and standard deviation (SD) of milk production in each DIM class.

**DIM class**	***N***	**Mean (kg)**	**SD (kg)**
5–34	28,704	23.7	7.45
35–64	37,991	26.6	7.79
65–94	41,787	26.5	7.92
95–124	43,506	26.0	7.89
125–154	44,033	25.1	7.89
155–184	44,781	24.4	7.93
185–214	45,873	23.7	7.99
215–244	45,688	22.9	8.04
245–274	44,373	22.0	8.10
275–305	42,831	21.4	8.20
Total	419,567	24.2	8.13

### Model

We used first to fifth order LPs (LP1–LP5) to fit random regression test-day models, respectively. The model equation was as follows:

(1) $$y_{i\,t\,k\,l\,m}=H\,Y\,S_{l}+A\,G\,E_{m}+D\,I\,M_{t}+\sum_{k=0}^{n\,r}z_{i\,t\,k}\,a_{i\,k}+\sum_{k=0}^{n\,p}z_{i\,t\,k}\,p\,e_{i\,k}+e_{i\,t\,k\,l\,m}$$

where *y*_*itklm*_ is the observation of cow *i* measured at time *t* within herd-year-season (*HYS*) subclass *l* and age (*AGE*) subclass *m*; *DIM*_*t*_ is the fixed effect of DIM in 301 classes from 5 to 305 days; *a*_*ik*_ and *pe*_*ik*_ are the *k*th random regression of additive genetic and permanent environmental effects for cow *i*, respectively; *z*_*itk*_ is the *k*th order of LPs for cow *i* measured at DIM *t*, nr and np denote the order of LPs (from one to five) for additive genetic and permanent environmental effects, respectively; and *e*_*itklm*_ is the random residual. LPs in this study were defined as follows (Kirkpatrick et al., 1990):

$$L\,P_{0}\,\left(x\right)=\frac{1}{\sqrt{2}},L\,P_{1}\,\left(x\right)=\sqrt{\frac{3}{2}}\,x,L\,P_{2}\,\left(x\right)=\sqrt{\frac{5}{2}}\,\left(\frac{3}{2}\,x^{2}-\sqrt{\frac{1}{2}}\right),$$

$$LP_{3}\left(x\right)=\sqrt{\frac{7}{2}}\left(\frac{5}{2}x^{3}-\sqrt{\frac{3}{2}}x\right),LP_{4}\left(x\right)=\sqrt{\frac{9}{2}}\,\left(\frac{35}{8}\,x^{4}-\frac{30}{8}\,x^{2}+\frac{3}{8}\right),\mathrm{and}L\,P_{5}\,\left(x\right)=\sqrt{\frac{11}{2}}\,\left(\frac{63}{8}\,x^{5}-\frac{70}{8}\,x^{3}+\frac{15}{8}\,x\right).$$

where *x* is the standardized DIM, calculated as $x=\frac{2\,\left(D\,I\,M-D\,I\,M_{m\,i\,n}\right)}{\left(D\,I\,M_{m\,a\,x}-D\,I\,M_{m\,i\,n}\right)}-1$, and *DIM*_*min*_ and *DIM*_*max*_ are the minimum and maximum DIM, respectively.

In this study, March, April, May, September, and October were defined as calving season one; June, July, and August as calving season two; and November, December, January, and February as calving season three, and there were 1891 herd-year-season classes in total. Calving age was classified into four levels: 20–23, 24–27, 28–31, 32 months or later. Residual variance was assumed to be either homogeneous or heterogeneous across lactation. For models with heterogeneous residual variances, residuals were divided into 10 classes (5–34, 35–64, 65–94, 95–124, 125–154, 155–184, 185–214, 215–244, 245–274, and 275–305 DIM) (Pereira et al., 2013). The heterogeneous residual variances were handled by putting different weights on residual variance for different periods of DIM. The weights were calculated as $w_{i}=\frac{\bar{v}}{v_{i}}$, where *v*_*i*_ is the residual variance of the *i*th DIM class (Table 2), and $\bar{v}$ is the mean of residual variances.

**TABLE 2:** Estimates of residual variance (*v*_*li*_)_1_ over different classes of DIM and LP when assuming heterogeneous residual variances.

**DIM class**	***v*_*1i*_.**	***v*_*2i*_**	***v*_*3i*_**	***v*_*4i*_**	***v*_*5i*_**
5–34	29.20	18.76	13.95	14.54	15.32
35–64	17.24	15.29	15.44	14.04	12.61
65–94	14.33	14.70	13.05	11.88	11.90
95–124	14.92	14.12	12.18	12.23	11.40
125–154	15.34	12.99	12.45	11.80	11.33
155–184	15.30	12.54	12.48	11.39	11.26
185–214	14.06	12.63	11.57	11.43	10.54
215–244	12.53	12.75	11.23	11.15	10.80
245–274	11.60	11.12	10.97	9.95	9.64
275–305	16.21	10.38	8.64	9.57	9.80
Total	16.07	13.53	12.20	11.80	11.46

*^1^*v*_*li*_ is the residual variance of ith class in lactation with LP of order l.*

Additive genetic variance for a particular DIM was calculated as $\sigma_{g_{k}}^{2}=z_{k}^{\prime }\,G\,z_{k}$, *z*_*k*_ is a column vector of LP coefficients at the *k*th DIM, and **G** is covariance matrix of additive genetic effect. Permanent environmental variance for a particular DIM was $\sigma_{p_{k}}^{2}=z_{k}^{\prime }\,P\,z_{k},$ matrix *P* is a covariance matrix of permanent environmental effect, and *z*_*k*_ is the same as above; EBV of a particular animal at a particular DIM was calculated as ${\text{EBV}}_{m\,k}=z_{k}^{\prime }\,a$, *a* is column vector of additive genetic random regression coefficients of a particular animal, and *z*_*k*_ is same as above; the EBV for the whole lactation was calculated as ${\text{EBV}}_{m\,305}=\sum_{t=5}^{305}{\text{EBV}}_{m\,k}$. The estimation of variance components and prediction of breeding values using different models were carried out by the DMU package (Madsen et al., 2014).

### Model Comparison

Models with different orders of LPs were compared using the following methods based on full and reduced data sets:

1. Akaike information criterion (AIC; Akaike, 1973) and Bayesian information criterion (BIC; Schwarz, 1978). AIC was computed as *AIC* = −2*logL* + 2*p*, where −2*logL* is the restricted maximum log likelihood value, *p* is the total number of parameters estimated in the corresponding model. Bayesian information criterion was computed as *BIC* = −2*logL* + *plog*(*n*), where −2*logL* and *p* are the same as defined in AIC, *n* is the difference between number of test-day records and the rank of the fixed effects design matrix (Strabel et al., 2005). For both AIC and BIC, a lower value indicates a better goodness of fit.
2. Pearson correlation coefficient and Spearman’s rank correlation coefficient between EBV of 305-day (EBV305) estimated based on data with (full data) and without (reduced data) the phenotypes of animals calving in the last year was used to assess the prediction accuracy and the similarity between the rankings of animals based on those two groups of EBV305.

## Results

### General Statistics of TD Milk

Table 1 shows the mean for TD milk yield in the different classes of lactation, which ranged from 21.4 to 26.6 kg with standard deviations from 7.45 to 8.20 kg. An increase in milk yield was found up to 53 DIM, followed by a gradual decrease until the end of lactation. Averaged over different classes, TD milk was 24.2 kg with a standard deviation of 8.13 kg in first lactation Holstein cows.

### Goodness of Fit

Table 3 presents the number of estimated parameters and model comparison based on values of AIC and BIC. Number of estimated (co)variances for random effects of models increased from 7 to 43 when increasing the order of LP from one to five. Overall, AIC and BIC tend to be in favor of more complex models with higher orders of LP. However, the differences of AIC and BIC values were smaller among LP3, LP4, and LP5 compared with those among LP1, LP2, and LP3 for homogeneous residual variance. Smaller differences were observed across different orders of LP for heterogeneous residual variance compared with those for homogeneous residual variance. Furthermore, models with assumption of heterogeneous residual variances received lower values of AIC and BIC compared with models with assumption of homogeneous residual variance, indicating better goodness of fit.

**TABLE 3:** Number of variance components estimated for additive genetic effect (nA), permanent environment effect (nPE), AIC, and BIC for model comparison.

	**Order**^3^	**nA**	**nPE**	**AIC**	**BIC**
HO^1^	LP1	3	3	1,757,636	1,757,712
	LP2	6	6	1,739,185	1,739,327
	LP3	10	10	1,732,188	1,732,418
	LP4	15	15	1,728,916	1,729,255
	LP5	21	21	1,726,882	1,727,352
HE^2^	LP1	3	3	1,725,349	1,725,426
	LP2	6	6	1,722,036	1,722,179
	LP3	10	10	1,720,665	1,720,894
	LP4	15	15	1,716,308	1,716,647
	LP5	21	21	1,710,956	1,711,426

*^1^HO denote assumption of homogeneous residual variance. ^2^HE denote assumption of heterogeneous residual variance. ^3^Random regression test-day model with Legendre polynomials (LP) of order *i* (*i* from 1 to 5).*

### Comparison on Estimated Variance Components and Heritability

**FIGURE 1:**
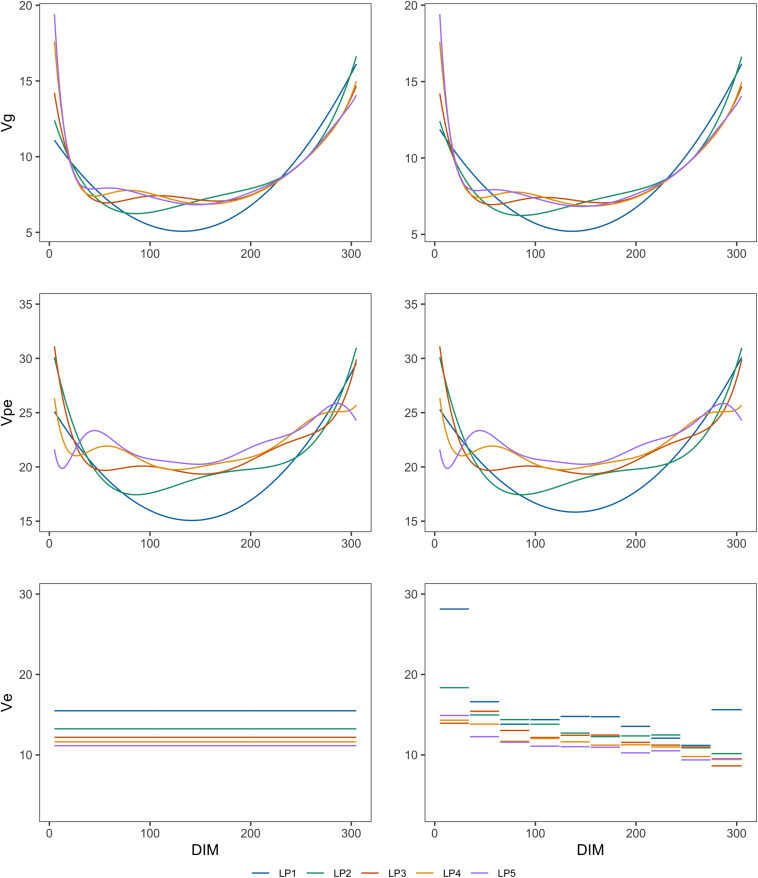
Genetic variances (Vg), permanent environmental variances (Vpe), and residual variances (Ve) at each test day along the lactation from models with different orders of LP based on assumption of homogeneous (left column) or heterogeneous residual variance (right column).

**FIGURE 2:**
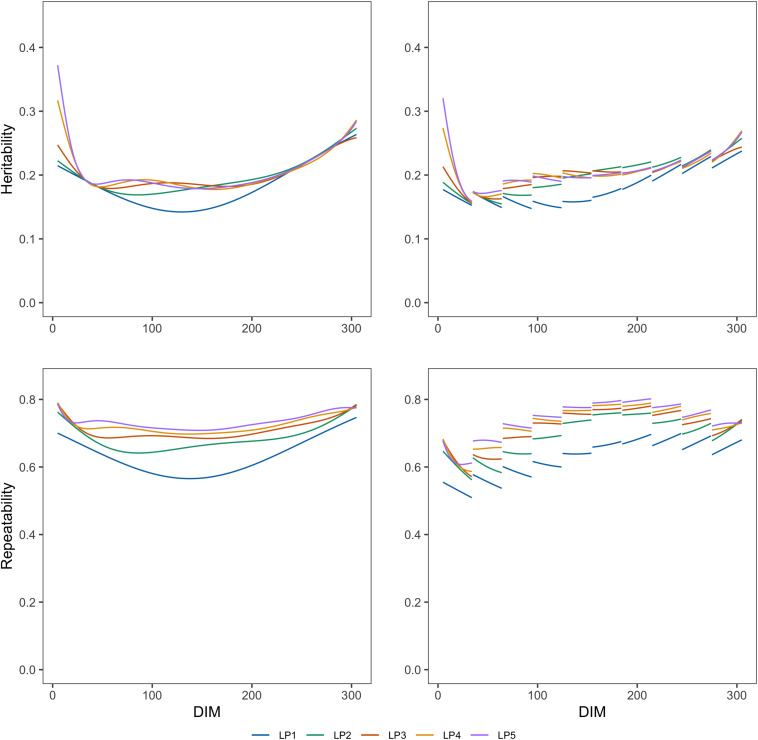
Heritabilities and repeatabilities at each test day along the lactation from models with different orders of LP based on assumption of homogeneous (left column) or heterogeneous residual variance (right column).

Figures 1, 2 show the genetic variances ($\sigma_{g}^{2}$), permanent environmental variances ($\sigma_{p}^{2}$), residual variances ($\sigma_{e}^{2}$), heritabilities (*h*^2^), and repeatabilities (*Rep*) calculated for each day along the lactation trajectory based on the estimated covariance function coefficients. The curve of genetic variances showed a sharp decrease in the early lactation and an increase from the middle to the end of lactation. The curve of permanent environmental variances presented similar trends as the genetic variances. Estimates of genetic and permanent environmental variance were slightly different in models with different orders because higher order had more inflexion points. For estimates of residual variance, fitting higher order of polynomial yielded a lower horizontal line when assuming homogeneity in the model. In addition, when assuming heterogeneity in the model, although the whole horizontal line was split into 10 segments, the same trend as the homogeneous model was observed with respect to the order of fit. In particular, peaks can be seen at the beginning of lactation when fitting lower orders of polynomials. Similarly, the heritability was high at the beginning and end of lactation and lower in the middle.

Repeatability decreased in early lactation and increased to the end of lactation when using the models with homogeneous residual variance although it decreased at the first stage of lactation and increased to about a DIM of 200, then decreased slightly to the end of lactation when using the models with heterogeneous variances.

Table 4 shows the heritability (*h*^2^) and repeatability (*Rep*) for 305-day milk yield and the minimum and maximum values for TD milk yield. *h*^2^ for 305 days estimated from models with different LPs ranged from 0.250 to 0.257, and *Rep* were from 0.741 to 0.749 when considering homogeneous variance. When considering heterogeneous variance, *h*^2^ for 305 days were between 0.250 and 0.260, and *Rep* were between 0.738 and 0.749. *h*^2^ for TD from models with different LPs ranged from 0.142 to 0.372 and *Rep* were between 0.566 and 0.786 when considering homogeneous variance. When considering heterogeneous residual variances, *h*^2^ for TD ranged from 0.143 to 0.326, and *Rep* were between 0.520 and 0.821. Models with LP1 and LP2 led to higher estimated heritability and lower repeatability than the models with higher order. Heritability and repeatability for 305 days milk yield estimated from the models with LP3, LP4, and LP5 were very similar, ranging from 0.249 to 0.250 for heritability and from 0.748 to 0.749 for repeatability. Models with homogeneous or heterogeneous residual variances led to similar estimates of heritability and repeatability except for the LP1 model.

**TABLE 4:** Heritabilities (*h*^2^) and Repeatabilities (*Rep*) for 305 days milk yield and minimal (Min) and maximal (Max) *h*^2^ and *Rep* for TD in different LP considering homogeneous (HO) and heterogeneous (HE) residual variances.

	**LP**	**HO**			**HE**		
		**305 days**	**Min for TD**	**Max for TD**	**305 days**	**Min for TD**	**Max for TD**
*h*^2^	LP1	0.257	0.142	0.264	0.260	0.143	0.269
	LP2	0.254	0.169	0.273	0.254	0.163	0.303
	LP3	0.250	0.179	0.259	0.250	0.163	0.304
	LP4	0.250	0.177	0.317	0.249	0.170	0.326
	LP5	0.250	0.179	0.372	0.249	0.171	0.303
*Rep*	LP1	0.741	0.566	0.747	0.738	0.520	0.773
	LP2	0.744	0.641	0.782	0.744	0.628	0.821
	LP3	0.748	0.685	0.788	0.748	0.639	0.821
	LP4	0.749	0.697	0.791	0.749	0.682	0.799
	LP5	0.748	0.708	0.786	0.749	0.688	0.799

### Comparison on Predictive Ability

Table 5 presents Pearson correlation and Spearman’s rank correlation coefficients between EBV305 from full and reduced data. In general, the highest correlation was observed from LP3. For both Pearson correlations and Spearman’s rank correlations, the correlation coefficients increased from LP1 to LP3 and kept relatively stable from LP3 to LP5 for both homogeneous and heterogeneous residual variances. Models with homogeneous and heterogeneous residual variances yielded similar prediction accuracies across different orders of LP. A similar pattern was found for the rank correlations.

**TABLE 5:** Pearson correlations (Spearman’s rank correlations) between EBV of 305-day milk yield obtained from full and reduced data for models with different orders of LP and with homogeneous (HO) or heterogeneous (HE) residual variance.

**Residual variance**	**LP1**	**LP2**	**LP3**	**LP4**	**LP5**
**HO**	0.703 (0.684)	0.713 (0.689)	0.731 (0.701)	0.730 (0.700)	0.729 (0.697)
**HE**	0.694 (0.682)	0.711 (0.688)	0.733 (0.702)	0.732 (0.702)	0.729 (0.698)

## Discussion

In this study, various criteria were used to compare random regression test-day models with different order of LPs. Different criteria for model comparison have been discussed by Druet et al. (2003) and Odegard et al. (2003). In our study, models with higher order obtained lower AIC and BIC values, which was in line with previous studies (Pereira et al., 2013). This means models with LP5 fit data best regardless of complexity. However, models with higher orders introduced more parameters and resulted in a heavier computational demand. Therefore, in practice, model selection needs to balance between goodness of fit and model complexity.

The further improvements of goodness of fit by increasing order of LP became smaller when using higher order of LP. In particular, when assuming homogeneous residual variance, there was a smaller improvement by increasing order of fit from LP3 to LP4 and from LP4 to LP5 based on values of AIC and BIC. Similar results were observed by Lopez-Romero and Carabano (2003) based on a Spanish Holstein population, in which the reduction in residual variance was small when increase order of fit from LP4 to LP6. In addition, they reported the order of LP3 was the optimum choice based on the BIC values although higher order (LP6) was needed for permanent environmental effects.

The trajectory of additive genetic variances and permanent environmental variances showed a decrease at the beginning of lactation and an increase at the end of lactation. A similar pattern was reported by Van Der Werf et al. (1998), Pool et al. (2000), and Strabel et al. (2005) in Polish, Dutch, and Australian Holstein populations. Higher additive genetic variances at the beginning and end of lactation might be attributed to variations in the number of TD records, milk yield level, or non-genetic factors, for example, pregnancy effects (Meyer, 2005). Other studies have also shown that fitting a higher order of LP produced higher estimates of genetic variances at the edges of lactation and an oscillatory pattern along the lactation trajectory, which might be unlikely biologically (Jamrozik et al., 1997a; Pool et al., 2000; Lopez-Romero and Carabano, 2003; Meyer, 2005). This indicates that a model with a higher order (e.g., LP5) may not be more optimal than a model with a lower order (e.g., LP3 or LP4).

Heterogeneous residual variances were generally observed over the lactation when analyzing test-day records in dairy cattle populations (White et al., 1999; Brotherstone et al., 2000; Jensen, 2001). Therefore, it is reasonable to overcome this impact. It can be clearly seen that the residual variances changed across 10 DIM groups defined in this study although the differences were small when fitting higher orders of LP. More specifically, the estimated residual variances were larger in the early lactation as also shown in other studies (Swalve, 1995; Jensen, 2001). In addition, Figure 1 reveals that the slope of the permanent environmental variance curve was affected by the change of residual variance over the lactation when assuming homogeneous residual variance in the model (Figure 1). This pattern can be explained by the fact that the permanent environmental effects have the ability to absorb part of the heterogeneity of the residuals (Ødegard et al., 2003).

In practical breeding programs, a critical procedure is to obtain accurate breeding values for the candidates via genetic evaluation and then make selection decisions based on the rankings of EBVs of the animals. Therefore, predictive ability is an essential property in these circumstances. In this study, predictive ability of the models was assessed by both Pearson correlations and Spearman’s rank correlations between EBV305 from a full and a reduced data set to evaluate the predictive abilities between models. A model with better goodness of fit does not necessarily indicate better predictive ability, whereas a poorly fit model, such as underfitting, induces bias. This was demonstrated by the highest coefficients of Pearson and Spearman’s rank correlation from models using LP of order three regardless of the assumption of residual variance. Hence, this suggests that fitting a model with order three of LPs is reasonable to achieve better predictive ability for milk yield.

## Conclusion

The results in this study show that goodness of fit increased with increased order of LP. The results demonstrate that further improvement became small when using models with order of LP higher than three. This indicates that a minimum of LPs of order three is necessary for estimating variance components and breeding value of milk yield. Considering a balance between goodness of fit, computational demand, and predictive ability, random regression test-day models using LP3 or LP4 appear to be the appropriate models for implementation of genetic evaluation in different Chinese Holstein populations.

## Author’s Note

This manuscript has been released as a pre-print at bioRxiv (Li et al., 2019).

## Data Availability Statement

The raw data supporting the conclusions of this article will be made available by the authors, without undue reservation.

## Ethics Statement

Ethical review and approval was not required for the animal study because The data are milk production of dairy cattle collected from dairy farms. Written informed consent was obtained from the owners for the participation of their animals in this study.

## Author Contributions

JL, HG, and GS conceived and designed the study. JL and HG conducted all analysis and wrote the draft. GS revised manuscript. PM maintained the DMU software package used in the statistical analysis. RL, WL, PB, GX, YG, and XD helped with the data collection and project coordination. All authors provided critical feedback and helped shape the manuscript.

## Conflict of Interest

XD was employed by the company Linqing Rutai Animal Husbandry Co., Ltd, Liaocheng, China. PB and GX were employed by the company Shandong OX Livestock Breeding Industry Co., Ltd, Jinan, China. The remaining authors declare that the research was conducted in the absence of any commercial or financial relationships that could be construed as a potential conflict of interest.
